# Supplementary material for: Two-Pore-Domain Potassium Channel TREK–1 Mediates Pulmonary Fibrosis through Macrophage M2 Polarization and by Direct Promotion of Fibroblast Differentiation
Source: Biomedicines. 2023 Apr 26;11(5):1279. doi: 10.3390/biomedicines11051279 (PMC10215911; doi:10.3390/biomedicines11051279)
Supplement: Supplementary file 1 [file biomedicines-11-01279-s001.zip › biomedicines-2345181-supplementary.pdf]

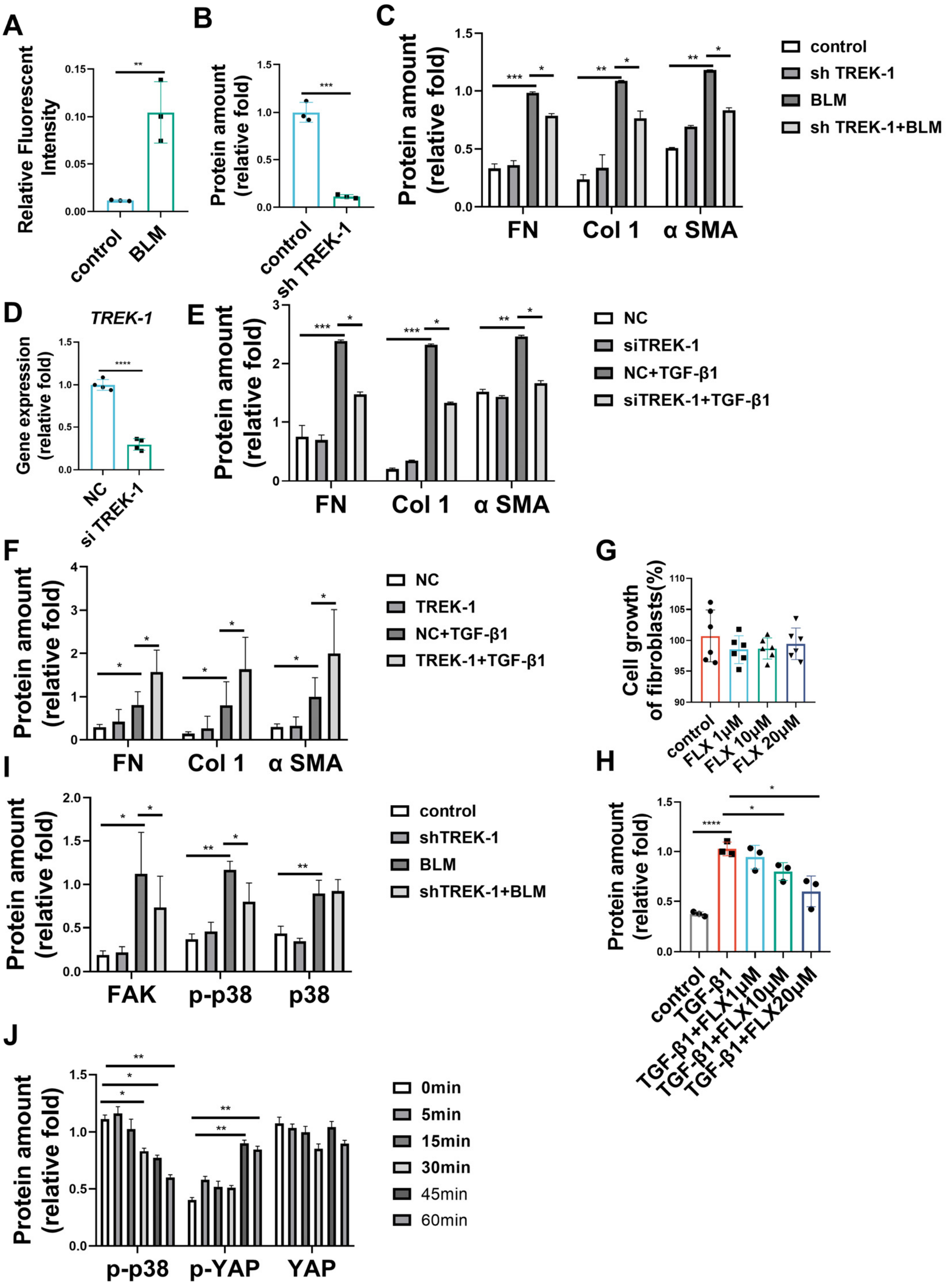

Figure S1. (A) Relative fluorescence intensity of TREK-1 in Figure 1E. (B) Semi-quantitative analysis of TREK-1 level evaluated by Western blotting in Figure 3A. (C) Semi-quantitative analysis of protein level evaluated by Western blotting in Figure 3C. (D) Interference efficiency of small interfering RNA in fibroblasts. (E) Semi-quantitative analysis of protein level evaluated by Western blotting in Figure 6C. (F) Semi-quantitative analysis of protein level evaluated by Western blotting in Figure 6D. (G) Cell viability of fluoxetine treated fibroblasts by CCK8 assay. (H) Semi-quantitative analysis of α-SMA level evaluated by Western blotting in Figure 6G. (I) Semi-quantitative analysis of protein level evaluated by Western blotting in Figure 6H. (J) Semi-quantitative analysis of protein level evaluated by Western blotting in Figure 6I.
